# Enabling Implementation of Competency Based Medical Education through an Outcomes-Focused Accreditation System

**DOI:** 10.5334/pme.963

**Published:** 2024-02-06

**Authors:** Timothy R. Dalseg, Brent Thoma, Keith Wycliffe-Jones, Jason R. Frank, Sarah Taber

**Affiliations:** 1 Department of Medicine, Division of Emergency Medicine, University of Toronto, Toronto, ON, Canada; 2 Royal College of Physicians and Surgeons of Canada, Ottawa, ON, Canada; 3 Toronto General Hospital, 200 Elizabeth Street, R. Fraser Elliott Building, Ground Floor, Room 480, Toronto, ON M5G 2C4, (416) 833-0121; Canada; 4 Department of Emergency Medicine, University of Saskatchewan, Saskatoon, SK, Canada; 5 Department of Family Medicine, Cumming School of Medicine, University of Calgary, Calgary, AB, Canada; 6 Centre for Innovation in Medical Education, University of Ottawa, Ottawa, ON, Canada

## Abstract

Competency based medical education is being adopted around the world. Accreditation plays a vital role as an enabler in the adoption and implementation of competency based medical education, but little has been published about how the design of an accreditation system facilitates this transformation. The Canadian postgraduate medical education environment has recently transitioned to an outcomes-based accreditation system in parallel with the adoption of competency based medical education. Using the Canadian example, we characterize four features of an accreditation system that can facilitate the implementation of competency based medical education: theoretical underpinning, quality focus, accreditation standards, and accreditation processes. Alignment of the underlying educational theories within the accreditation system and educational paradigm drives change in a consistent and desired direction. An accreditation system that prioritizes quality improvement over quality assurance promotes educational system development and progressive change. Accreditation standards that achieve the difficult balance of being sufficiently detailed yet flexible foster a high fidelity of implementation without stifling innovation. Finally, accreditation processes that recognize the change process, encourage program development, and are not overly punitive all enable the implementation of competency based medical education. We also discuss the ways in which accreditation can simultaneously hinder the implementation of this approach. As education bodies adopt competency based medical education, particular attention should be paid to the role that accreditation plays in successful implementation.

## Introduction

The implementation of competency based medical education (CBME) throughout Canadian postgraduate medical education (PGME) has resulted in an unprecedented level of reform. With the College of Family Physicians of Canada (CFPC) implementing its Triple C Competency-based Curriculum and the Royal College of Physicians and Surgeons of Canada (Royal College) transitioning to Competence by Design (CBD), the way in which trainees are educated and assessed in PGME has fundamentally changed [1,2,3]. Implementation of CBME is a large-scale, complex change initiative [4]. Without successful implementation, learners will fail to benefit from the purported advantages of this educational approach.

While others have characterized the CBME implementation experience and have identified factors perceived to have influenced the outcome, the literature fails to discuss the important role that accreditation plays in enabling this transition [5,6,7]. The substantive changes that have taken place in the Canadian PGME system have occurred in parallel with a transition to an outcomes-based accreditation process within PGME. There must be alignment between both the educational paradigm and the accreditation system, or the implementation of transformational change will be hampered.

We describe how a unique, collaboratively constructed accreditation system enabled the transition to, and implementation of, CBME. The lessons learned are shared to facilitate the continuous improvement process locally and to inform the world discourse on accreditation as a tool to maximize fidelity and integrity of implementation in these evolving systems.

## Definitions

Competency-based medical education is “oriented to graduate outcome abilities, organized around competencies derived from an analysis of societal and patient needs” (p. 636) [8]. Individual learner competencies are observable abilities that integrate knowledge, skills, and attitudes [9]. CBD is the Royal College’s version of CBME that began implementation in 2017, and combines an outcomes-based approach to learning while utilizing time as a resource [3]. CBME is more than a curriculum. It involves the design, implementation, assessment, and evaluation of the associated educational program as well as a series of policy decisions and implications [9,10]. The adoption of CBME, and in this case CBD, has been characterized as a transformational change, requiring a significant shift in behaviours [11].

Accreditation is “the process of formal evaluation of an educational program, institution, or system against defined standards by an external body for the purposes of quality assurance and enhancement” (p. 4) [12]. The fit-for-purpose framework for accreditation systems states that the best design prioritizes local needs and contexts, resulting in a tailored approach that is adaptable to a jurisdiction’s changes over time [13]. By prioritizing contexts, the fit-for-purpose framework establishes an important connection between local factors and an accreditation system’s ability to effectively conduct a situationally meaningful accreditation in its pursuit of quality. One such local context that must be considered in the design of any accreditation system is the educational approach: in this case, CBME [13,14].

### CanERA

In Canada, three colleges share a mandate to accredit PGME: the CFPC, the Collège des Médecins du Québec (CMQ), and the Royal College. The colleges’ system of accreditation went without transformational change for decades (from approximately 1990 to 2010), with incremental improvements focused on enhancing standards and improving alignment between the colleges. The impetus for the current change arose in the late 2000s (circa 2008–2009), with key postgraduate medical education stakeholders signalling the need for modern redesign [15]. This call for change coincided with early developments of CBME models and the drive toward CBME in Canada [16,17].

**Figure 1:**
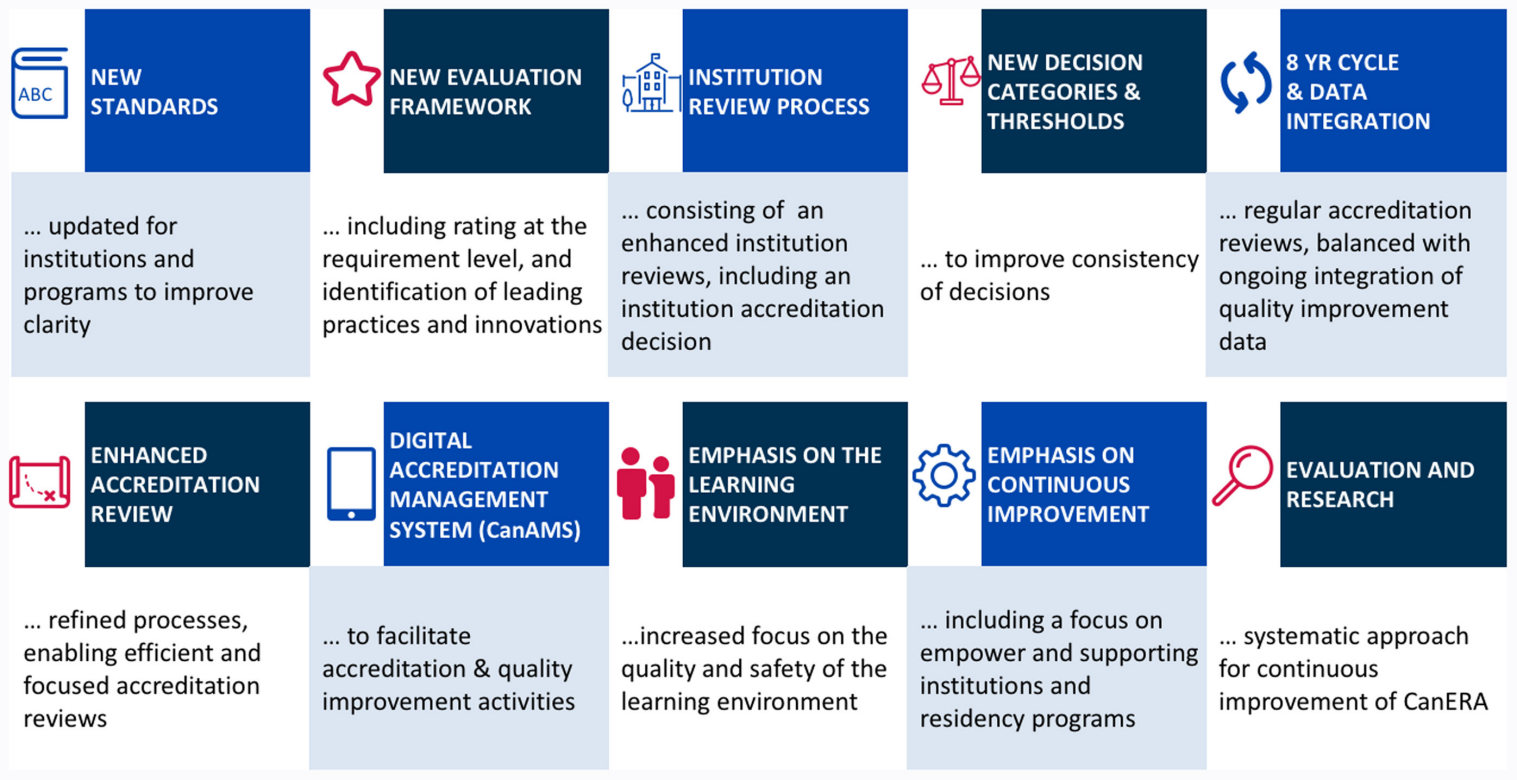
Transformational changes in the Canadian Excellence in Residency Accreditation (CanERA) accreditation system. Copyright 2017. The Canadian Residency Accreditation Consortium (CanRAC). https://www.canrac.ca/canrac/about-e. Reproduced with permission.

In response to this call for change, the three colleges formed the Canadian Residency Accreditation Consortium (CanRAC) in 2013. Collaboratively, CanRAC hosted a series of series of summits from 2013 to 2018 that included stakeholders from across the Canadian PGME and accreditation landscape. Throughout this process, CanRAC sought to identify opportunities for alignment and coordination of accreditation processes across the continuum of medical education, incorporate and innovate on best practices in accreditation, and adapt to changes taking place within the education paradigm [18]. This work culminated in the launch of a new, unified PGME accreditation system entitled *Canadian Excellence in Residency Accreditation (CanERA)* officially on July 1, 2019. CanERA features [18] a bundle of transformational accreditation changes (Figure 1).

A set of goals was established for CanERA with the design and launch of this new system. These included an intent to; modernize the approach to accreditation through new standards, provide a new framework to guide standards’ evaluation, increase the emphasis on continuous quality improvement, reduce workload for all stakeholders through a longer eight year accreditation cycle and a digital accreditation management platform, and to support CBME implementation through standards, policies, and processes that align with the principles of CBME.

## What features of an accreditation system enable the implementation of CBME?

### Alignment of educational theory

Previous work has proposed that accreditation systems and their design are influenced by the environments in which they are situated [13]. However, the opposite is true as well: the educational environment can be profoundly influenced by the accreditation system and its design. It must be recognized that accreditation systems wield considerable influence through their regulatory mandates. Potential accreditation outcomes drive the educational behaviour and decisions of those being accredited. When implementing a new educational paradigm, it is therefore important to have an accreditation system that promotes and prioritizes shared characteristics and constructs. Alignment of the underlying features, including the theoretical basis of the new paradigm, educational priorities, and societal responsibilities, is critical to ensure that implementation is driven in the direction of intended educational change.

Traditional accreditation systems were focused on structures and processes, which was well suited to an educational system that relied on time to ensure that learners had received the required training to be a competent physician. As an outcome-based educational paradigm, CBME utilizes a curriculum that is organized around the assessment, documentation, and interpretation of outcomes in the form of competencies across the educational experience [9,19,20]. CanERA’s new accreditation system design reflects a departure from the more easily evaluated constructs of structure and process and instead places emphasis on outcomes within the accreditation standards [21]. For example, instead of simply requiring that a policy exists, those undergoing accreditation would be expected to demonstrate how a policy is used, and its outcome, to establish compliance with the standard. Within an outcomes-based accreditation system, postgraduate training programs are more freely able to adopt and prioritize educational innovations that are consistent with the principles of CBME. These may include educational interventions, learning experiences, and assessments that are focused on the outcomes of the trainee. For example, instead of requiring that all learners rotate through a specific educational experience, programs are free to utilize non-traditional, or learner-driven experiences to satisfy educational competencies. The ability to incorporate these types of critical components is important to advancing the fidelity of implementation or, in other terms, the extent to which the critical components of an innovation are present in an enacted system [22].

Furthermore, the principles of an outcomes-based accreditation system align with the educational outcome priorities of CBME on a more fundamental level: specifically, an emphasis on graduate and program outcomes that will create competent physicians who will contribute to, and be responsible for, improving the health of individuals and populations [9,23,24]. This alignment serves to strengthen the validity of the accreditation system and process, while simultaneously addressing expectations for social accountability [9,23,24]. Alignment at this level increases the likelihood that programs and institutions will integrate and apply these essential principles in their own contexts, thereby furthering the integrity of implementation [25].

### Quality assurance versus quality improvement focus

Systems of accreditation have a purpose that often includes both quality assurance and quality improvement [12]. A quality assurance focus assures the public, regulators, and others that programs meet minimum standards for educational quality thereby preventing harm, particularly during a period of major curricular reform [26]. Accreditation organizations thus have an important fiduciary duty to institutions and programs, trainees, and, ultimately, to patients. Conversely, a quality improvement focus emphasizes helping programs and, ultimately, the system of medical education, to improve quality through the conduct of self-evaluation and achieve aspirational standards over time all while having a lower risk to their accreditation status [27,28]. Arguably, this notion of pursing improvement may be particularly important during the early stages of CBME adoption. A quality improvement philosophy can also help to promote the identification and sharing of “next” and best practices and thus promote the diffusion of innovation through a period of CBME implementation [27]. While some accreditation systems place greater emphasis on one quality objective over the other, most systems find themselves situated somewhere between these two ends of a spectrum [13].

To meet both priorities, the CanERA system reframed its mandate and placed increased emphasis and expectations on continuous quality improvement while retaining design features that focused on quality assurance. At the program level, this emphasis was manifested by the creation of a new quality improvement domain within the accreditation standards [29]. This domain, with its associated standards and indicators, was created to ensure that a culture of quality improvement was present in postgraduate training programs and to clearly define the expectations to which all programs would be held. In this new system design, a balance was struck that advanced implementation while maintaining safety within the system [29].

### Accreditation standards

Accreditation standards can be defined as “measures or generally accepted benchmarks used in making decisions about the quality of a program, institution, or system” (p. 6) [12]. Thus, standards outline the expectations for programs’ and institutions’ achievement of quality and drive associated behaviour to meet the criteria [30,31]. Accreditation standards are perhaps one of the most fundamental components of an accreditation system [32]. In the case of CBME, ensuring that the content of accreditation standards is aligned with and ideally based upon the core components of a competency-based design [11] will help to drive the implementation of CBME.

Accreditation standards differ in terms of their level of detail, the flexibility afforded in their requirements, and their focus on structures, processes, and/or outcomes. Accreditation standards that are highly detailed or prescriptive, or that primarily emphasize structural and process-based criteria, may be most effective at driving standardization across programs [33]. In CBME, this can provide clarity to programs in terms of expectations and drive fidelity in relation to the desired model of educational design. However, it may be argued that this approach could also stifle important innovation that occurs through the natural processes of change diffusion [34]. Conversely, standards that are written to allow for greater flexibility or that place a greater emphasis on educational outcomes may facilitate programs’ abilities to innovate in their local implementation of CBME. This approach, combined with a mechanism that allows the accreditation standards to be continuously informed by innovations as implementation occurs, could be quite powerful in driving ongoing iteration and improvement, thus supporting the implementation of CBME. However, overly flexible or vague standards may not provide programs with the guidance they need, particularly at early stages of implementation, and may ultimately result in a loss of fidelity of implementation of the desired model of educational design.

Overall, the CanERA standards were written with a balance of structure, process, and outcome measures in mind, reflecting a spectrum from the more detailed to the more flexible, respectively. Specifically, the standards were structured in a hierarchy, beginning with an overarching standard, under which is nested elements, requirements and indicators; the evaluation framework requires each measurable requirement and indicator to be evaluated [29], providing precise feedback to programs about what has been implemented according to the standards’ expectations and about where areas for improvement are identified. For example, the standard regarding the educational program (standard 3) states an overarching outcome that, “Residents are prepared for independent practice,” (p.9) [29]; its nested elements, requirements and indicators then outline expectations based on the core components of CBME. The specific indicators in this standard that are then evaluated through the accreditation process provide specific guidance to programs in terms of what is required as part of CBME implementation (e.g., Indicator 3.1.1.2: “The competencies and/or objectives address each of the Roles in the CanMEDS/CanMEDS-FM Framework specific to the discipline” (p.9) [29]) and where more flexibility in how to achieve the outcome is afforded (e.g., 3.2.3.1: “Individual residents’ educational experiences are tailored to accommodate their learning needs and future career aspirations, while meeting the national standards and societal needs for their discipline” (pg.9) [29]). The content of CanERA standards, combined with their intentional evaluation structure and wording, act as a powerful enabler in the transition to CBME.

### Accreditation processes

“Accreditation processes” is a broad term used to encompass several core activities common to most accreditation systems, including how the standards are evaluated (i.e., models of self-assessment and external assessment, information used to evaluate standards), how decisions are rendered (i.e., decision categories and processes), by whom (i.e., site review models), and how often (i.e., the accreditation cycle) [13]. There is significant variation in these processes across accreditation systems. What is important — or perhaps essential — is that they are aligned with and reward (and therefore promote) the desired behaviours of the program or institution being accredited [13].

The CanERA accreditation system introduced several features intended to help foster the implementation of CBME. First, programs were given time to work on CBME implementation before any accreditation impacts. Specifically, where the accreditation standards require that programs implement CBD according to specialty-specific educational requirements, for example the requirement for programs to use specialty-specific learner competencies in defining objectives for the program (indicator 3.1.1.1, pg. 9 [29]) or specific educational experiences, e.g., rotations (indicator 3.2.1.2, pg. 9 [28]), the accreditation policies ensured that programs would not be expected to comply with those requirements for the first year of a specialty’s CBD launch [35].

Second, CanERA introduced new approaches to accreditation decision-making intended to support CBD implementation. CanERA accreditation categories and associated decision-making principles were revised to encourage and reward continuous improvement rather than being punitive; specifically, the decision principles were designed such that programs and institutions that actively share with accreditors what they are working on are not penalized for those areas at the time of the accreditation decision, provided they achieve minimum standards and are making improvement [35]. This is contrasted with previous accreditation models where any gap in the standards, including those known to the program where improvements were being made, were included in the program’s formal list of weaknesses, and factored into the accreditation decision. Additionally, the newly introduced accreditation decision at the institution level, and additional standards requirements for how institutions oversee their postgraduate training programs and assist them with quality improvement, is intended to bolster the role that PGME offices and their leadership play in leading an institution-wide change such as CBME.

Finally, the new CanERA accreditation process aims to foster innovation and experimentation, particularly with novel approaches to successfully implement CBD. In CanERA, a leading practice and innovation (LPI) is defined as “a practice (method, procedure, etc.) that is noteworthy for the discipline, or residency education writ large; and/or is *unique and innovative* in nature” [35]. LPIs introduced the ability for surveyors to recognize programs and institutions for a novel or interesting practice that could be of interest to other programs or institutions across the country. The aim is to share these practices in the future, including novel and successful approaches to implementing CBD, in a database that other programs looking for ways to address specific areas for improvement or generally improve their program could access.

## How accreditation can simultaneously be a barrier to implementation of CBME

While accreditation can act as an enabler of the implementation of CBME, features of the system, standards, and processes introduce limitations and consequences.

Despite agreement that the aligned focus on outcomes within both accreditation and CBME can enable implementation [36,37], there are challenges that come with an over-reliance on outcomes in accreditation [23]. In any curricular enterprise, there are elements, including educational processes and features of the learning environment, that cannot be evaluated in the form of outcomes [24] and yet are inherently important to the quality of the program and therefore to accrediting bodies. Rigid adherence to the outcomes-based construct would prevent these elements from being effectively evaluated, compromising the fiduciary responsibility that accreditation bodies hold. Furthermore, the absence of these constructs within the accreditation system design could impair early implementation through deficient characterization of these required elements (see Table 1).

**Table 1:** How features of an accreditation system can enable or hinder implementation of competency based medical education.


ACCREDITATION SYSTEM FEATURE	ENABLE IMPLEMENTATION OF CBME	HINDER IMPLEMENTATION OF CBME

Alignment of educational theory	Alignment of educational theory within the educational paradigm and accreditation system drives change in a consistent and intended direction. Example: Outcomes-based alignment: a decreased emphasis on structure and process in accreditation facilitates the adoption of CBME principles within programs.	Rigid adherence to a single educational theory ignores the importance of competing traditional constructs preventing their evaluation and risk impairing early implementation. Example: Even in an outcomes-based system, process elements such as attributes of the learning environment are not best evaluated by outcomes, yet remain critical to effective curricular implementation.

Quality focus	A quality improvement focus drives self-evaluation, with an objective of system development that can facilitate adoption of CBME, driving the fidelity and integrity of implementation. This focus also promotes sharing of best practices and diffusion of innovations.	A quality assurance focus ensures that minimum standards are met by programs, minimizing the risk of harm to stakeholders. In doing so, programs prioritize the achievement of standards instead of the implementation of CBME. Adoption of innovations is not prioritized.

Accreditation standards	Highly detailed and prescriptive standards can provide clear expectations, improving the fidelity of implementation. Outcome-based standards that allow greater flexibility promote innovation and site-specific adaptations that encourage implementation.	Highly detailed and prescriptive standards can stifle innovation and the change process, therefore impairing implementation. Outcome-based standards may be too flexible, resulting in inadequate guidance and a low fidelity of implementation. Standards that fail, or are slow to evolve, may slow the pace of CBME adoption.

Accreditation processes	Leniency on accreditation standards during the first year of implementation may support programs’ transition to CBME. Accreditation decision categories and decision-making principles that promote continuous quality improvement within programs can facilitate implementation without punitive consequences. Recognition of novel leading practices and innovations can foster implementation by other programs.	The perception of a lower stakes accreditation process through leniency and tailored decision-making principles may, for some, reduce the motivation for change and CBME implementation.


*Abbreviation: CBME:* competency based medical education.

Accreditation is, by nature, a cyclical process spanning many years; programs often spend several years preparing to meet accreditation standards, which can make it challenging for accreditation systems to be agile and ensure standards remain relevant [34]. Yet, to promote the implementation of CBME, certain accreditation standards may be aspirational or, at a minimum, be flexible enough to support the desired changes. Anachronistic standards that conflict with the desired model of CBME, such as prescriptive requirements regarding time spent in training, risk unduly punishing programs that adopt CBME and slowing the overall pace of CBME adoption and implementation across the system. Accreditation bodies thus have a duty to ensure their standards keep pace with major changes in medical education — in this case, CBME.

Finally, the resource implications of accreditation reform alongside CBME change can present a barrier. As existing systems of accreditation evolve to better match the adoption of the CBME paradigm, many internal changes are required to maintain the quality and effectiveness of the accreditation process [36]. The exact resource requirements for large-scale changes such as these have not been described but, as an example, Greenfield et al. [38] noted that the development and revision of accreditation standards for general practice in Australia alone was a significant, challenging, and difficult undertaking requiring considerable resources. These implications could be even greater when the accreditation process itself is novel or significantly modified because of the adoption of CBME. Changes to an accreditation system have the potential to then result in resource implications that cycle back to the program. Engagement and participation in an accreditation process has resource impacts that sometimes stretch over a period of many months to years. This has the potential for negative impacts not only on the educational mission that the accreditation process is there to examine but also on the implementation process of CBME.

## Considerations for future system design

CBME represents a fundamental shift in medical education design to emphasize outcomes, primarily the competencies acquired by graduates of a given program. In the future, methods and technologies developed to provide intelligence based on program activities and graduate outcomes may transform the accreditation enterprise.

1. Program evaluation analytics. CBME programs generate large amounts of assessment data that, in addition to informing trainee progression [39,40], could provide insights into other aspects of the program that are the focus of accreditation standards such as the teaching and assessment practices of faculty [41] and rotations [42]. This information could provide particularly valuable insight into the effectiveness of quality improvement efforts that are driven by the accreditation process [43], as faculty development efforts would be expected to lead to improved supervisor feedback of trainees on assessments and changes to rotation schedules to improve the alignment between the desired and realized completion of assessments. The use of this type of data could therefore advance the specificity and objectivity of evaluation processes in accreditation.
2. Program outcomes standards. Beyond the inclusion of simple assessment metrics, the opportunity to track more distal educational and clinical outcomes could provide an opportunity to evaluate additional dimensions of quality from an accreditation lens that would not be possible without the adoption of CBME [4,20,44]. For example, what better evidence of a quality educational program could there be than the demonstration that graduates of a program have appropriate clinical outcomes once they begin practice? The robust integration of educational and clinical data can, at the program level, provide insight into how well a residency program is preparing its graduates for practice [45] while at the system level it could provide insight into features of residency training that result in graduates with better clinical outcomes. These insights could, in turn, inform the development of future accreditation standards.
3. Validity evidence for transformative designs. Finally, given the high-stakes nature of the accreditation process and the value that is attributed to it, it would be interesting to consider how the validity argument for accreditation [46] might be strengthened as new accreditation designs roll out in tandem with CBME [47]. The search for validity evidence to support medical accreditation and the decisions that are a part of it are not new [48]. Current published validity evidence is limited and typically restricted to the validity of unique survey tools and face validity of isolated standards [48,49]. The transformative design decisions within CanERA described above were made to not only enable the implementation of CBME, but to advance the quality of decision making that is part of accreditation. While anecdotal evidence would suggest that this has been successful, assessment data, program analytics, and new program outcome measures could all be used in the future to explore this objectively and contribute to the validity of accreditation decisions.

## Conclusion

Accreditation systems have a powerful role to play in enabling the transition to CBME. Their features, including their alignment of underlying educational theory, quality focus (quality assurance versus quality improvement), accreditation standards, and accreditation processes, all represent design opportunities that can be used to facilitate this transition. It must be acknowledged however, that within each of these domains there are limitations and that all design opportunities can come with a consequence. We have presented examples from the CanERA accreditation system to illustrate the opportunities and limitations that have been observed to date, as well as the opportunities for the future. Educational institutions that are considering or are undertaking a transition to CBME should consider the importance of accreditation in enabling the successful transition to this new educational paradigm.

## Disclaimer

The views and opinions expressed in this article are those of the authors and do not necessarily reflect the official policy or position of the Royal College of Physicians and Surgeons of Canada (“Royal College”). Information in this article about Competence by Design (“CBD”), its implementation and related policies and procedures do not necessarily reflect the current standards, policies and practices of the Royal College. Please refer to the Royal College website for current information.
